# Being in the zone during physiological birth: a comparative study of hospital and home birth environments

**DOI:** 10.3389/fgwh.2025.1573688

**Published:** 2025-04-15

**Authors:** Orli Dahan, Alon Goldberg

**Affiliations:** ^1^ Department of Multidisciplinary Studies, Tel-Hai College, Kiryat Shmona, Israel; ^2^ Department of Education, Tel-Hai College, Kiryat Shmona, Israel

**Keywords:** physiological birth, birthing consciousness, birth environment, flow state, home birth, hospital setting

## Abstract

**Introduction:**

A flow experience typically occurs when the challenge of a demanding physical activity aligns with an individual's abilities, resulting in a sense of empowerment and fulfillment. Experiencing flow during physiological childbirth occurs in various birth environments, but quantitative studies comparing home birth and hospital birth in this respect are scarce. Childbirth is a psychological, social, and physiological event; thus, the birthing environment probably crucially affects the mental state of birthing women. We hypothesized that home birth will be positively correlated with a heightened flow state experienced by women during physiological labor, differing significantly from the experience of women birthing in a hospital.

**Method:**

Israeli women with physiological childbirth experience were recruited through social media. Participants (*n* = 421) completed the Flow State Scale (FSS) and a demographic questionnaire.

**Results:**

Comparing hospital births and home births, our research reveals a significant correlation between home birth environment and heightened birthing women's flow state. In physiological childbirth, women birthing at home report higher flow states compared to women in hospitals.

**Discussion:**

The observed differences indicate a compelling connection between the birthing environment and the women's experience during labor. The heightened flow state during home births is explained in measured flow dimensions: challenge-skill balance, action-awareness merging, clear goals, unambiguous feedback, concentration, and joy. By comparing correlations of birthing environments and birthing women's flow state, this research contributes a novel perspective to the ongoing discourse on optimizing childbirth experience.

## Introduction

Hospital birth is the cultural norm in Western industrialized countries, and the percentage of planned home births, for example, in the US, remains relatively low (1). This study took place in Israel, where maternity care operates within a general healthcare system, ensuring all residents have access to comprehensive prenatal, childbirth, and postnatal services (2). Most births in Israel occur in hospitals, accounting for approximately 99% of all births (3). These hospitals offer a spectrum of childbirth options, ranging from highly medicalized births with interventions like epidurals to low-intervention births supported by midwives (3, 4). Israeli midwives are registered nurses with specialized midwifery training (3). They provide autonomous care and emotional support, often managing multiple births simultaneously (4). Obstetricians, however, hold ultimate responsibility for birth outcomes and intervention decisions, sometimes influenced by liability issues. Thus, it is a medicalized maternity care system (3).

Planned home births are legal in Israel but remain relatively uncommon, constituting approximately 1% of all births (3, 5). These births are typically attended by certified midwives and are subject to strict regulations to ensure the safety of both mother and baby. Thus, women opting for home births must meet low-risk criteria (6). As for birth centers, in 2017, the Israeli Ministry of Health ordered the closure of all independent, midwife-led birth centers. This decision significantly reduced the options for out-of-hospital births (6, 7). However, in July 2021, the High Court ruled that these closures violated women's freedom of choice, ordering the reopening of these centers. Despite this ruling, free-standing birth centers have not yet resumed operations (7).

Qualitative studies reveal why women choose to plan home birth. Women's past traumatic encounters with hospital births, coupled with a desire for a more natural birthing process, appear to have driven their confident choice to opt for planned home births despite facing criticism and social stigma from both their social circles and certain maternity care providers (8, 9). In this context, an important example of a positive physiological outcome of home birth is reduction in perineum trauma. Perineum trauma severely affects postpartum women's ability to recover both physiologically and psychologically from birth, and planned home births are associated with fewer perineal tears and a reduced risk of third or fourth-degree tears during childbirth (10). Additionally, women who birth at home are less prone to experiencing postpartum depression compared to those who give birth at a hospital or birthing center (11). Women who opt for a planned home birth also report a high level of satisfaction, attributing it to the comfort of their home environment and the sense of increased control over the birthing experience. Qualitative studies consistently highlight feelings of empowerment during home births as a prevalent theme (12).

A positive birth experience is of paramount importance for both the mother and the newborn. It sets the stage for a healthy start to life, fostering immediate bonding and successful breastfeeding (13). A positive experience can also significantly impact the mother's emotional well-being, reducing the risk of postpartum depression and anxiety (14). It empowers women, making them feel confident and in control, which can have long-lasting effects on their self-esteem (15). Research shows that women view a positive birth experience as involving the safe birth of a healthy baby in a supportive environment, with both practical and emotional assistance from birth companions and understanding clinical staff. While many prefer physiological birth, most women recognize the uncertainty of childbirth and are willing to adapt. If intervention is required, women aim to retain a sense of control through active involvement in decision-making. In this sense, both safety and psychosocial wellbeing hold equal importance (14).

Conversely, negative birth experience usually correlates with a more medical birth, particularly instrumental births and unplanned cesarian sections (16), lack of support during childbirth (17), and obstetric violence (18). Negative birth experiences may have severe mental consequences during postpartum, such as childbirth-related PTSD or symptoms of somatization, obsessive compulsion, depression, and anxiety (16); thus, according to the World Health Organization, healthcare providers and support systems should strive to ensure that every birth is a positive, empowering experience (19). While there is a growing emphasis on promoting positive birth experiences, recent research by Kuipers et al. (20) explored women's firsthand accounts of their childbirth experiences across seven European countries – United Kingdom, Netherlands, Belgium, Germany, Austria, Spain, and the Czech Republic. The study specifically examined women's perceptions of their role and treatment within maternity care systems, revealing persistent issues of marginalization. Despite residing in countries often viewed as progressive and woman-centered, participants frequently reported not being treated as equal partners or primary decision-makers during their births (20).

Recent studies have demonstrated that during a physiological birth, women sometimes experience a positive altered state of consciousness (13). This state is referred to as “birthing consciousness,” and it resembles the mental flow state that is characterized by complete immersion and focus on an activity (4, 21). In a previous report, we discussed differences identified in an online survey of 766 women regarding the flow state experienced during childbirth. The findings showed that women who underwent physiological childbirth (i.e., without epidural anesthesia or instrumental interventions) had a higher flow state during birth (21). The flow state, often experienced during intense physical activity, involves focused engagement with a task that is both physiologically and psychologically challenging (22). During flow, individuals experience intense concentration, a sense of timelessness, effortless action, and deep enjoyment. Flow often occurs when the challenge of an activity matches the individual's abilities, leading to optimal performance and a feeling of accomplishment (23–27). This description fits fundamental aspects of physiological childbirth, thus labor and birth can induce a profound state of flow (28). It is crucial to note that flow experiences do not necessarily imply superficially cheerful sensations; rather, they involve a profound sense of intrinsic motivation, fulfillment, and positive engagement with the task at hand (29). Thus, experiencing flow during childbirth does not mean women feel explicitly cheerful or conventionally joyful throughout the entire labor, but rather that they experience labor as intrinsically meaningful, rewarding, and empowering.

According to Kirkham (28), while many women experience stressful births in medicalized birth environments, others seek autonomy in their birthing experience. These women often opt for home births with midwife care, valuing continuity, trust, and empowerment. A Flow state can occur in various birth settings, including traditional maternity wards, if women are treated with respect and actively involved. These experiences boost maternal confidence and strength (28). Thus, experiencing flow during physiological childbirth occurs not only in domestic birth environments – such as birth centers and homes – but also in typical hospital settings, but few studies compare the two. Because childbirth is a psychological, social, and physiological process, the birthing environment probably crucially affects the mental state of birthing women, especially during unmedicated birth (13, 30), we assumed the possibility of different subjective birthing experiences based on environment. The goal of our study was to investigate this potential link between birth environment and the physiological childbirth experience based on the occurrence of a heightened flow state during childbirth. For purposes of the study, we defined physiological birth simply as a vaginal birth with no epidural and no instrumental assistance in the second stage (see also (16).

### Research hypothesis

Home birth will be positively correlated with a heightened flow state experienced by women during physiological labor, differing significantly from the experience of women birthing in a hospital environment.

## Methods

### Procedure

Israeli women were invited through social media to participate in an open online study presented as “Experience during physiological childbirth.” The physiological mode of birth was defined simply as vaginal birth without an epidural or instrumental assistance in the second stage of birth. In the current study, we intentionally chose to focus solely on the type of birth – specifically, physiological birth defined as birth without epidural analgesia or instrumental assistance during the second stage – as our primary criterion of interest. We deliberately refrained from collecting additional data regarding other birth interventions, such as the frequency of vaginal examinations or various forms of labor induction. This decision was made to maintain a concise, clear, and accessible online questionnaire, thereby maximizing participant responsiveness and reducing dropout rates.

After signing informed consent forms, each woman received a link and was asked to complete separately the online demographic questionnaire and a flow state questionnaire. Participants were informed that their anonymity would be preserved throughout the study and that they had the right to discontinue participation at any time. There was no financial incentive for participating; we did offer to share the results of the study with the participants. Respondents were able to review and change their answers through a Back button.

### Instrument

#### Demographic questionnaire

We collected information about participants' age, marital status, number of children, education level, number of years since the birth being reported, number of births, birth order of the selected birth (first, second, etc.), and the environment in which their physiological birth took place: hospital or at home.

#### The Flow State Scale (FSS)

The FSS is a 36-item self-report questionnaire that assesses flow experiential awareness in sports and physical activity settings, which was developed based on Csikszentmihalyi (22) and is well-validated (25). The scale uses nine dimensions to evaluate flow awareness: challenge-skill balance (e.g., “I felt I was competent enough to meet the high demands of giving birth”), action-awareness merging (e.g., “I did things spontaneously and automatically”), clear goals (e.g., “I knew clearly what I wanted to do”), unambiguous feedback (e.g., “I could tell by the way I was performing how well I was doing”), concentration on the task (e.g., “I had total concentration”), sense of control (e.g., “ I felt in total control of my body”), loss of self-consciousness (e.g., “I was not worried about what others may have been thinking of me”), transformation of time (e.g., “Time seemed to alter – either slowed down or speeded up”), and autotelic experience (e.g., “I found the birthing experience extremely rewarding”). Each item was rated on a 5-point Likert scale ranging from 1 (strongly disagree) to 5 (strongly agree). Mean internal consistency of the sub-scales in the current research was .87–.95.

While the Flow State Scale (FSS) was originally designed to measure flow experiences during demanding physical activities, it was adapted for this study to assess flow during childbirth. The instructions for completing the FFS questionnaire stipulated that the woman should answer the questions regarding the specific physiological childbirth experience she chose for the demographic questionnaire. Minor adjustments were made to certain items to align them more closely with the specific context of childbirth, such as replacing “high demand of the situation” with “high demands of giving birth.” This adaptation allowed for the application of the FSS to the unique physiological and psychological aspects of childbirth, providing a more nuanced exploration of flow within this context [see Supplementary Appendix A: Adapted Flow State Scale (FSS) for Childbirth Context].

### Ethical considerations

Ethics approval was obtained from the Institutional Review Board (12/2021-7).

## Results

### Participants

Participants were 421 Israeli women who had experienced physiological childbirth. Of the women, 305 (72.4%) gave birth at the hospital and 116 (27.6%) gave birth at home. Their mean age was 36.42 years (*SD* = 7.34). The mean number of years after the reported birth event was 4.56 (*SD* = 3.11), with 42.5% of the women reporting on their first childbirth, 26.8% their second childbirth, and 30.6% other births. Demographically, 93.1% had a graduate/professional degree; and 64.4% were secular while 35.6% were traditionally religious.^1^

We have posted our invitation to participate in the survey on various women's Facebook groups, such as “Mothers on Maternal Leave” and “Natural Birth & Home Birth.” The latter group is a private group of nearly 40,000 women. This can explain the relatively high percentage of women who reported experiencing homebirths (27.6%), while in Israel, only 1% are giving birth at home.

### Preliminary results

To test whether the demographic variables correlated with research variables, a series of Pearson correlation tests were conducted between the woman's age, number of years since reported childbirth, number of children and FSS scores. Furthermore, Spearman correlation tests were conducted between education level, number of births, birth order of the selected birth (first, second, etc.), religiosity and FSS. Finally, differences in demographic variables between women who birthed at home vs. women who birthed at hospital were tested. Results demonstrated no significant associations between demographic variables and research variables.

### Research hypothesis analyses

To examine the hypothesis on the differences between physiological home childbirth and physiological hospital childbirth, a MANCOVA analysis was conducted with the place of childbirth as the independent variable, flow state level as the dependent variable, and mother's age, child's age, and birth number as covariates. Results demonstrated that the MANCOVA model was significant [*F* (9,399) = 4.18, *p* < .0001]. ANOVA tests revealed significant differences between the place of childbirth groups for most flow state dimensions, with women who experienced home childbirth reporting higher flow states than women who experienced physiological childbirth at the hospital (Table 1). The results confirmed our hypothesis.

**Table 1:** MANOVA analysis: differences between the place of physiological childbirth on Flow State Scale (*N* = 421).

	Home childbirth (*n* = 116)		Hospital childbirth (*n* = 305)		
Flow State Scale	*M*	SD	*M*	SD	*F* (1,407)
Challenge-skill balance	4.60	.53	4.45	.72	4.38*
Action-awareness merging	4.14	.85	3.67	.93	22.65***
Clear goals	4.32	.76	4.07	.93	6.46*
Unambiguous feedback	4.15	.85	3.86	.98	7.88**
Concentration	4.44	.68	4.02	.85	23.25***
Sense of control	3.64	1.14	3.43	1.10	2.66
Loss of self-consciousness	4.37	.75	4.18	.95	4.03*
Transformation of time	3.72	1.09	3.57	1.10	1.41
Autotelic experience	4.22	1.02	3.90	1.05	9.42**

* *p* < .05.

** *p* < .01.

*** *p* < .0001.

## Discussion

We emphasize that childbirth is a complex and multifaceted event characterized by significant individual (31) and cultural (32) variability among birthing women. The findings of this study are limited in scope, focusing specifically on the reported experience of flow during physiological birth in home settings compared to physiological birth in hospital environments. Thus, the results should not be generalized broadly to all births or other birthing contexts.

Women who had physiological births at home reported elevated levels of mental flow state for 7 of the 9 dimensions of flow, indicating a correlation between birthing environment and the birthing woman's experience during labor. Several interconnected factors differentiating between the home and hospital environments explain the connection to each flow dimension. An in-depth explanation of each flow dimension can enrich our understanding of the subjective physiological birth experience – a perspective unexplored in the literature to date.

Moreover, recent theoretical advancements in flow research reinforce the significance of studying flow within natural, dynamic settings rather than controlled laboratory environments. Durcan et al. (33) expand the understanding of environmental and perceptual factors shaping flow experiences, emphasizing the role of autonomy, motivation, and personal connection to the activity. Their critique of traditional research approaches, which rely on sterile, short-term tasks in artificial settings, highlights the need for models that capture flow in real-world contexts. Similarly, the ecological dynamics perspective (34) underscores the dynamic interplay between individuals and their environment, proposing that perception, action, and environmental affordances are integral to the emergence of flow. While these studies do not address childbirth specifically, their insights align with our finding that the physical and social birth environment play a central role in shaping flow experiences during birth. The contrast between hospital and home birth provides a unique opportunity to explore how differences in autonomy, privacy, medical interventions, and environmental design influence flow states. Applying this approach to childbirth research strengthens the argument that factors such as physical design, emotional support, and birth setting are not peripheral but central to the flow experience. By investigating flow in birth environments rather than experimental settings, this study contributes to a growing body of literature advocating for a broader valid understanding of flow in high-intensity, real-world experiences.

Home settings offer a sense of comfort and familiarity that can positively impact a woman's experience during labor. Women feel they can have a customized birth experience at home but not in an institutionalized setting (35). Being in a familiar environment reduces stress and anxiety, which are known to hinder the progression of labor. The woman feels more relaxed, which contributes to the process of physiological birth (36). Reducing anxiety and pain during labor can also reduce the length of the first stage of labor (37). Indeed, in a qualitative study on the reasons of Canadian women for planning a home birth, the women outlined that laboring at home provided greater flexibility in pain management and coping strategies (35). Moreover, in a home birth setting, women typically have more autonomy regarding their birthing process (38). They have the freedom to move around, choose positions that feel most comfortable, and make decisions about their care in collaboration with their birth attendants. This sense of autonomy enhances feelings of agency, leading to a more positive childbirth experience (13).

These characteristics of home environment in contrast to the typical hospital environment can explain the flow dimensions of *unambiguous feedback* (“I had a good idea while I was birthing about how well I was doing”), *action-awareness merging* (“I made the correct movements without thinking about trying to do so”), and *challenge-skill balance* (“I felt I was competent enough to meet the high demands of giving birth”). The shared feature of these dimensions is the enhanced mind-body connection. Feeling autonomy to move at will and being free to use any strategies to ease the pain of contractions and feel comfortable in her surroundings allows for more profound connection to her body (13) and, in a way, letting her body to do its job without the mind interfering (36). After all, while childbirth is a demanding physiological process, it is very much affected by psychological factors such as comfort and confidence (15). Indeed, women who planned home birth voiced a preference for home birth not only for its comfort but also for the increased autonomy it offered (35).

The enhanced mind-body connection, expressed through automatic bodily movements and spontaneous actions during physiological birth, aligns with previous findings in the literature. Hrdy (39), xii–xiv) similarly emphasized that labor contractions may be perceived by women as intense yet captivating biological forces, evoking fascination rather than mere distress or pain (see also (40–42). These insights correspond closely to the current study's findings on the flow experience, reinforcing the idea that physiological birth uniquely facilitates embodied awareness and a relinquishment of conscious control, ultimately enriching the overall birth experience.

In this context, choosing a home birth reflects a deep trust in the body's ability to give birth naturally (43). Because having a home birth is not very common in industrialized societies, women who opt to plan for home birth must take ownership of their decision (35). Hence, it could be said that the sense of ownership – belief in the ability to give physiological birth – starts before birth itself. The sense of empowerment of women following physiological birth is a known phenomenon in midwifery literature (15). It is sometimes referred to as “the superwoman syndrome”: The euphoric sensation of accomplishment and joy that some women experience post-birth due to the surge of hormones and the sense of empowerment that comes with successfully giving birth (15, 42, 44). This feeling can lead to a heightened sense of confidence and capability, as if the woman can conquer anything, like a superhero. It reflects a deeply positive and empowering experience of childbirth that can leave a woman feeling invincible and capable of taking on any challenge (15). These profound sensations of euphoric pleasure can explain the flow dimension of *autotelic experience* (“I really enjoyed the experience of giving birth”). Thus, the emotional and practical meaning of the differences in the flow state scores can be translated into an enhanced positive peak experience at home.

Home birth settings provide a more intimate and private environment where birthing women can labor surrounded by loved ones and supportive birth attendants. This intimate setting positively impacts the woman's emotional state and overall experience of labor (45). For most people, home is a peaceful and restful place. A hospital setting is much different in environment and culture, and hospital staff have certain routines that can affect the birthing process. It is not uncommon to have different people walk in and out of a laboring woman's room. In one's own home, people who enter are invited guests and are usually individuals who will provide the woman with good support (12). This issue of privacy is highly crucial when it comes to physiological birth and is related to at least two dimensions of flow: *loss of self-consciousness* (“I was not concerned with how I was presenting myself”) and *concentration on the task* (“I was completely focused”).

During intense contractions, women report that it is helpful to handle the pain by focusing and retreating to a different zone, which also helps to relinquish some social constraints (36). The ability to be unconcerned about what others might think is crucial, especially during intimate and vulnerable events, such as sexual activity or giving birth (13). According to Cohen Shabot and Korem (18), the bodies of birthing women during physiological unmedicated childbirth are oxymoronic. When women give birth, from a functional point of view they complete one of the most fundamental missions of femininity – to bring new life into the world. However, at the same time, their bodies are the complete opposite of what society views as feminine: birth involves mess, blood, noisy sounds, and pain. A birthing body during physiological birth is a strong, expanding, loud, messy body; it challenges femininity's usual frame. The event of physiological birth is blatantly sexual in its “inappropriate” way. The norms of femininity are not to be overly sexual or exuberant – but to be beautiful and self-controlled, silent, delicate, obedient (18). Thus, when a woman is able to overcome the perceived “lost dignity” in physiological birth (46) she becomes uninhibited and can relax, which, in turn, can promote the birth process that feelings of embarrassment often hinder (13). Privacy and support from people who are not strangers – during one of life's most intimate episodes – can be crucial to focusing and surrendering self-consciousness (21, 44).

Home births involve fewer medical interventions than hospital births because the commonly used interventions in hospitals (oxytocin drips, epidurals, c-sections) cannot be implemented at home births. But as for other interventions, such as routine vaginal examinations, homebirth midwifes hold a philosophy of birth that is woman-centered. This philosophy is also a physiologic care model that emphasizes supporting and advocating for physiological childbirth, employing medical intervention only when necessary (47). As a result, birthing women are less likely to experience unnecessary interventions such as continuous fetal monitoring (48), or routine vaginal examinations (49, 50). Avoiding these interventions, or even the need to refuse to interventions, can contribute to the elevated sense of flow experienced by home birthing women by letting them focus on their goals – on what is important to them, to their specific situation, and not to general, strict hospital protocols. This feature can be related to the flow dimension of *clear goals* (“I knew what I wanted to achieve”), which reflects the strong feeling of confidence of the birthing woman.

In this context, Neerland et al. (45) suggest that midwives' trust in the natural childbirth process and their belief in its normalcy help instill similar confidence in birthing women. Conversely, many hospital settings have a different dynamic, where women report achieving physiological birth despite pressures from medical staff to accept unnecessary interventions (51). Although many midwives in hospitals aim to practice woman-centered care, rigid protocols, and a medicalized hospital philosophy can hinder such care, creating external pressures for interventions that may not always be medically necessary. As a result, women may feel compelled to advocate for their preferences and goals, sometimes facing provider-centered rather than woman-centered approaches. For example, birthing women often express the desire for empowerment, autonomy, and control over their birth experiences rather than being perceived or treated as weak or incapable (35).

As explained in the introduction, this study is about home births and not birth centers because all birth centers in Israel were closed in 2017 by the Israeli Ministry of Health see (52). However, birth centers are like home birth in many respects, such as being domestic birth environments with a women-centered philosophy and a tendency to promote a physiological birth. Thus, our findings here support prior studies of birth centers, which were found to increase feelings of confidence among birthing women (45). Birth centers, with intimate, homelike environments, are calming, fostering feelings of ease and empowerment during childbirth. The welcoming atmosphere of birth centers also contributed to birthing women's satisfaction. The design of birth rooms can affect the birthing experience. For instance, in birth centers, unlike in typical hospital birthing rooms, safety equipment is not prominently displayed. This contributes to the heightened confidence of birthing women (45), perhaps even allowing them to focus on their goals without interruption.

Two dimensions were not found to be elevated for home environments: the *sense of control* (“I felt in total control of what I was doing”) and the *transformation of time* (“Time seemed to alter”). A plausible explanation for the absence of differences in the sense of control dimension between home and natural hospital births may lie in the inherent complexity and ambiguity of the notion of control during physiological birth. As previous qualitative syntheses illustrate (53), women's psychological experiences during physiological childbirth are characterized by a paradoxical interplay between actively maintaining control and simultaneously surrendering or relinquishing it. Achieving a physiological birth requires women to feel empowered and in control of their decisions and environment, yet also demands the capacity to let go of conscious control to allow the physiological processes of birth to unfold naturally. This nuanced and ambivalent experience of control might explain why our study did not detect significant differences between home and hospital settings, given that the subjective meaning and control experience transcend straightforward categorizations by birth environment.

The sensation of time alteration is not unique to a positive peak experience; people can experience alterations in time perception (the feeling that time either slowed down or sped up) in relatively negative experiences such as simply being bored, or in the case of childbirth, during highly negative and traumatic experiences (54). Thus, it is reasonable that the event of birth would be experienced with a blurred sense of time, regardless of the mode of birth or the setting in which it took place (see also (21). Qualitative research will be more effective in measuring these more nuanced, specific sensations – i.e., the sense of control and the sensation of time alteration – during the experience of birthing.

## Conclusion

In physiological childbirth, women birthing at home report higher flow states than those giving birth in hospitals. Given that the flow experience represents a highly positive and beneficial psychological state, it is important to further understand how it can be facilitated across different birth settings. Considering that the majority of women today give birth in hospital settings, future research should explore how conditions associated with increased flow experiences can be effectively integrated into hospital-based care, enhancing women's overall birth experiences.

### Limitations and strengths

It is well established that the birthing environment significantly influences birth outcomes, including the mode of birth and the psycho-physical consequences that impact recovery and postpartum mental health. The current study employed a cross-sectional design; hence, there are limitations to any conclusions regarding causality realtionships between birthing at home and a hightened flow state during physiological childbirth. However, this study's significance lies in its novel perspective on measuring positive physiological birthing experiences. It also lies in being the first to identify a link between the strength of this experience and the domestic setting compared to the experience of the same birthing mode in a typical hospital environment. This is also one of the few studies applying the quantitative method to examine the physiological birth experience in terms of flow.

A potential limitation of this study could be recall bias, given the significant time elapsed (median = 4.56 years) between childbirth and data collection. However, childbirth represents a significant milestone with lasting psychological and emotional implications for women (55). It marks a profound transitional phase in life, carrying potential for both empowerment and trauma, influenced by factors such as personal perceptions of the birth experience, method of birth, and the availability of continuous support (56–58). Research dating back to Simkin (59) illustrates that women retain clear, detailed memories of their childbirth experiences even long after the event. Contemporary studies further validate these findings, demonstrating that due to childbirth's transformative nature, the experiences associated with it remain exceptionally vivid in women's long-term memory (60). Our current study focuses on physiological birth experiences, which are generally perceived as more positive than highly medicated births (13, 61). Therefore, despite the time elapsed since birth, the vividness and emotional intensity of these memories likely mitigate potential bias, reinforcing the reliability of women's retrospective accounts.

Our analysis did not control all labor interventions because we defined physiological birth as non-instrumental vaginal birth without epidural analgesia. This could include a range of interventions that may interrupt the physiological process and that would not occur if the labor and birth were happening spontaneously (“naturally”), including episiotomy (in both settings) or labor induction (via membrane sweep in both settings and by pharmacological means in hospital). This means that the differences found in the flow state could also be about the level of interventions. While this analysis could not be done for the current study, given that labor and birth procedures were not recorded, other explanations for the findings exist, and future studies that collect more information could test these alternatives. Numerous other factors not explored in our study – such as prior expectations, the extent of social and professional support, and previous birth experiences—may significantly influence reported flow experiences. Therefore, caution is warranted in interpreting these findings, acknowledging the phenomenon's complexity and highlighting the need for further research to investigate additional explanatory factors underlying differences in flow experience among birthing populations. Our survey was conducted in Hebrew; thus the sample was limited to Hebrew speaking women. A future study could conduct comparative research across countries with diverse healthcare systems and birth practices to examine the influence of cultural and contextual factors on the flow state experience during childbirth in various physiological birth settings, such as hospitals, birth centers, and homes.

## Data Availability

The raw data supporting the conclusions of this article will be made available by the authors, without undue reservation.

## Footnotes

^1^In the survey, religiosity was assessed by self-report, with participants categorizing themselves as either “secular” or “traditionally religious.” In the Israeli context, “traditionally religious” generally refers to women who identify with Jewish religious customs and traditions to varying degrees, ranging from moderate observance to more strictly religious lifestyles, but not explicitly adhering to the stricter Orthodox definitions.
