# Supplementary material for: Being in the zone during physiological birth: a comparative study of hospital and home birth environments
Source: Front Glob Womens Health. 2025 Apr 15;6:1573688. doi: 10.3389/fgwh.2025.1573688 (PMC12037561; doi:10.3389/fgwh.2025.1573688)
Supplement: Supplementary file 1 [file Datasheet1.docx]

**Being in the Zone during Physiological Birth: A Comparative Study of Hospital and Home Birth Environments**

# Appendix A.

**Adapted Flow State Scale (FSS) for Childbirth Context**

The following is the adapted version of the Flow State Scale (Jackson & Marsh, 1996), modified for use in the context of physiological childbirth. Minor wording adjustments were made to reflect the physical and psychological characteristics of labor and birth. Participants were instructed to recall their most recent physiological birth and rate how much each statement reflected their experience, using a 5-point Likert scale ranging from 1 (strongly disagree) to 5 (strongly agree).

**Instructions**
Please recall your most recent physiological birth experience, and rate the extent to which each statement describes your experience:

1. I was challenged, but I believed my skills would allow me to meet the challenge.
2. I made the correct movements without thinking about trying to do so.
3. I knew clearly what I wanted to do.
4. It was really clear to me that I was doing well.
5. My attention was focused entirely on what I was doing.
6. I felt in total control of what I was doing.
7. I was not concerned with what others may have been thinking of me.
8. Time seemed to alter (either slowed down or speeded up).
9. I really enjoyed the experience.
10. My abilities matched the high challenge of giving birth.
11. Things just seemed to be happening automatically.
12. I had a strong sense of what I wanted to do.
13. I was aware of how well I was performing.
14. It was no effort to keep my mind on what was happening.
15. I felt like I could control what I was doing.
16. I was not worried about my performance during birth.
17. The way time passed seemed to be different from normal.
18. I loved the feeling of my performance during birth and would like to feel it again.
19. I felt I was competent enough to meet the high demands of giving birth.
20. I performed automatically.
21. I knew what I wanted to achieve.
22. I had a good idea while I was giving birth about how well I was doing.
23. I had total concentration.
24. I had a feeling of total control.
25. I was not concerned with how I was presenting myself.
26. At times, it almost seemed like things were happening in slow motion.
27. After giving birth, I felt great about the experience.
28. The challenge of birth and my abilities were both at a high level.
29. I did things spontaneously and automatically, without having to think.
30. My goals were clearly defined.
31. I could tell by the way I was performing how well I was doing.
32. I was completely focused on the task at hand.
33. I felt in total control of my body.
34. I was not worried about what others may have been thinking of me.
35. Sometimes it almost felt like time stopped.
36. I found the birthing experience extremely rewarding.
